# Macro- and Microscopic Analyses of Anatomical Structures of Chinese Gallnuts and Their Functional Adaptation

**DOI:** 10.1038/s41598-019-41656-6

**Published:** 2019-03-26

**Authors:** Qin Lu, Hang Chen, Chao Wang, Zi-xiang Yang, Pin Lü, Ming-shun Chen, Xiao-ming Chen

**Affiliations:** 1 Research Institute of Resource Insects, Chinese Academy of Forestry, Kunming, 650224 China; 2 The Key Laboratory of Breeding and Utilization of Resources Insects of State Forestry Administration, Kunming, 650224 China; 3 Forest University of Southwestern China, Kunming, 650224 China; 4 Kansas State University, Kansas, 66506 USA

## Abstract

The galls induced by *Schlechtendaia chinensis, Schlechtendaia peitan* and *Nurudea shiraii* on *Rhus chinensis* and gall induced by *Kaburagia rhusicola rhusicola* on *Rhus potaninii* Maxim. are the largest plant galls and have great economic and medical values. We examined the structures of galls and their functional adaptation using various macro- and microscopic techniques. The highly adapted structures include a stalk at the base that is specialized for mechanical support and transport of nutrients for aphids, and a network of vascular bundles which accompanying schizogenous ducts arranged in a way to best support aphid feeding and population growth. There are many circular and semicircular xylems traces in an ensiform gall in cross sectional views, which would provide more nutrition and occupy less space. We infer the evolution trail was flower-like gall, horned gall, circular gall and ensiform gall. And the possible evolutionary trend of the gall was bigger chamber, more stable mechanical supporting, easier for exchanging substance and transporting nutrients.

## Introduction

Galls are abnormal outgrowths of plant tissues induced by gall-inducing organisms, which included various parasitic insects and mites. It is estimated that there are ~4700 different species that can induce gall formation under certain conditions^1^. The induction of galls is believed to be a result of plant manipulation by gall-inducing agents, and because of this, galls are generally considered as extended structures of the gallicolous organisms^2–5^. Galls exhibit a great deal of variations in morphology, sizes, and wall structures^6^. The morphology of galls are affected by plant types, tissue types, gall-inducing agents, and environmental factors, and can be spherical, bursiform, bullet-shaped, flower-shaped, cylindrical, diamond and so on^7–11^ Galls can be located at almost everywhere on a plant, including roots, leaf bases, branches or leaflets of host plants. In addition to the variation in morphology, the internal structures of galls exhibit great diversity as well. Some galls have very simple structures such as galls formed by simply outgrown and curved leaf-tissues. Some galls have highly differentiated, hierarchical structures with multi-chambers, which contain collenchyma tissues, parenchymatous tissues, physalides-parenchymatous tissues, and nutritive cellular layers^12–14^.

So far, we have very limited information on the structures and functions of many types of galls. It is generally thought that the magnitude of cell enlargement and tissue outgrowth determine the sizes, shapes and structures of galls, whereas the composition and structures of hyperplastic plant tissues determine the functions^2,3,15^. The formation of galls is considered as a compromise outcome between two interacting species during long-term coevolution^4,5,16,17^. Host plants can minimize stress by sacrificing a small portion of plant tissues for gall formation. Gall-inducing parasites can obtain nutrition from galls as well as gain a shell of protection from natural enemies. Consequently, galls are generally differentiated as nutritive tissues inside and defensive tissues outside^10,11^. There are arguments regarding to which functions of galls are the most important for gall-inducing parasites among scholars. Some scholars think the most important function of galls is to offer gall-inducing parasites the protection against hostile environments and predators, whereas other scholars think the most important function of galls is to provide gall-inducing parasites nutrients via nutritive cells and nutrient sink^8^.

Among the gall-inducing organisms, aphididae-induced galls are estimated to represent 10–20%^1^. Galls induced by aphids contain highly fortified cell wall, which can be tenfold thicker than that in normal plant tissues, and the surface of the galls is usually covered with trichomes^17^. Inside aphid galls, there is plenty of parenchyma connected to phloem and abundant vascular bundles and laticiferous tubes. However, no dense tissues or nutritive cellular layers were found inside aphid galls. This type of gall structures is thought to be an adaptation to insects with sucking mouthparts such as aphids^12,18,19^.

Among the gall-inducing aphids, *Schlechtandalia chinensis* (Bell), *Schlechtandalia peitan*, *Kaburagia rhusicola rhusicola*, and *Nurudea shiraii* are the most important gall inducers because their galls have great economic and medical values. Biologically, one of the most interesting phenomena of these gall-inducing aphids is the rapid and dramatic expansion of aphid populations within a short time period in individual galls. When an aphid feeds on a host plant, it can promptly induce the formation of a gall^20^. Within a gall, thousands of aphids can be produced from a single aphid parent, called fundatrix, within about 4 months^21^. There are many interesting questions remain to be answered. For example, (1) How can individual galls provide sufficient nutrition for a rapid and dramatic population expansion of aphids? (2) Are there specialized gall structures that provide favorable environments for aphid population expansion? (3) What are the differences among the different galls? We choose four shapes of gall: horned-gall, ensiform gall, circular gall and the flower-like gall which were widely used in forestry production in china. The objective of this study is illustrate the differences of four type galls in their anatomical structure by examine the localization, inner and outer surface of the gall, connecting networks of galls, and analyze potential connections between structures and gall functioning.

## Results

### Host plant, localization, and morphology

The *Rhus chinensis* and *Rhus potaninii* were small deciduous tree, the height of trees were more than 10 m in the late stage, and the trees could induce the gall from three years old to death.

**Figure 1:**
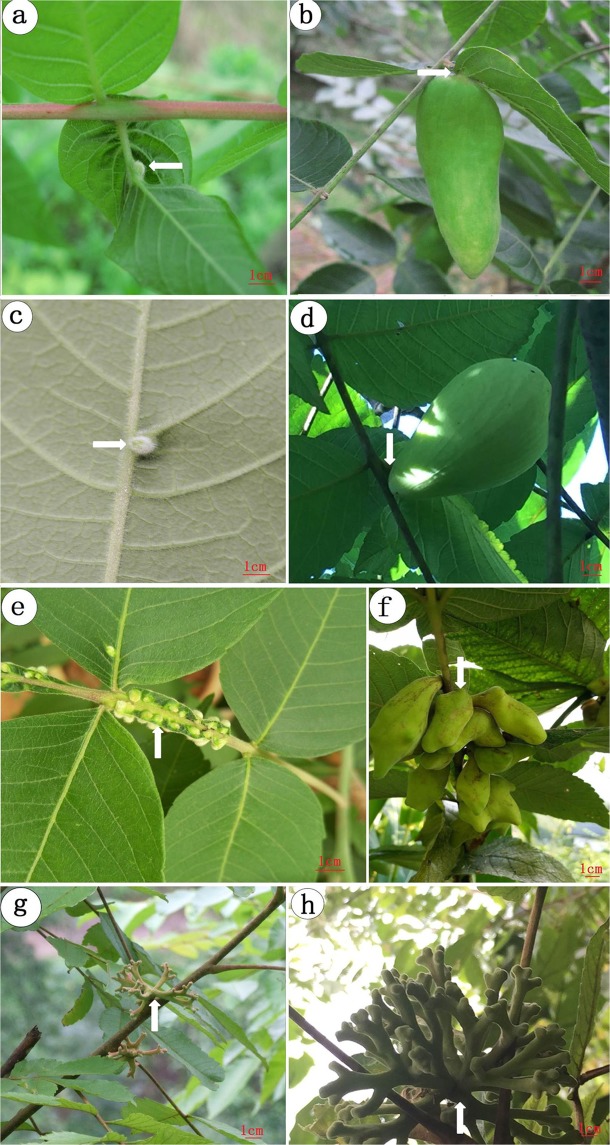
Localization and morphology of different types of galls. (**a**) A ensiform gall on a leaf of *R. potaninii Maxim* at the initial stage. (**b**) A mature ensiform galls on leaf blades. (**c**) A circular gall on a leaf at the initial stage. (**d**) A mature circular gall on a leaf blade. (**e**) multiple horned galls on a rachis wing at the initial stage. (**f**) Multiple mature horned galls on a rachis wing. (**g**) Flower-like galls on a rachis at the initial stage. (**h**) Mature flower-like galls on a rachis.

Figure 1 shows four types of galls induced by different aphid species. Representative galls of each type were shown at the initial stage (left panels) and mature stage (right panels). Each aphid species induces galls on different host plants, at different locations of the same host plant, or galls of different morphology. *S. chinensis, S. peitan*, and *N. shiraii* induce galls on *S. chinensis*, whereas *K. r. rhusicola* induces galls on *R. potaninii*. *S. chinensis* induces the formation of galls on minor rachis wings instead of major leaves (Fig. 1e,f). Because of the horned-shape of mature galls, *S. chinensis*-induced galls are also called horned galls or Chinese gallnuts. Multiple horned galls are usually clustered on individual rachis wings. On the other hand, both *S. peitan* (Fig. 1c,d) and *K. r. rhusicola* (Fig. 1a,b) induce long ovi-shaped mature galls on the main veins of major leaves. Galls induced by *S. peitan* are called circular horned galls and those induced by *K. r. rhusicola* are called ensiform galls. *N. shiraii* induces gall formation on the axillary bud (Fig. 1g,h), with galls consisting of many branches. Therefore, galls induced by *N. shiraii* are also called flower-like galls (Fig. 1g,h).

We counted the galls which grown on the different positions or different trees, the results were regular. The 99.01% of horned galls grown on the rachis wings, only 0.99% on the leaves. 100% of the circular galls grown on the leaves (*Rhus chinensis*) and 100% ensiform galls were induced in leaf (*Rhus potaninii)*. The total flower-like galls were induced in axillary bud (Table 1).

**Table 1:** The position of the galls.

	horned gall (%)	circular gall (%)	ensiform gall (%)	flower-like gall(%)
rachis wing (*Rhus chinensis*)	99.01%	0	0	0
leaf (*Rhus chinensis*)	0.99%	100%	0	0
leaf (*Rhus potaninii)*	0	0	100%	0
axillary bud (*Rhus chinensis*)	0	0	0	100%

More than 40 trees were counted of each type. *P < 0.05.

### Gall wall thickness

The thickness of gall wall differed among the different gall types. Mature ensiform galls showed the thickest wall, with 3.15 ± 0.41 mm (n = 45, *P* < 0.05). The wall of circular gall was 2.01 ± 0.24 mm (n = 45, *P* < 0.05). The wall of horned galls was thinner (1.82 ± 0.23 mm, n = 45, *P* < 0.05) than both circular horned gall and ensiform galls. The thinnest gall wall was flower-like galls, which was 0.67 ± 0.10 mm (n = 45, *P* < 0.05).

### The structures on outer and inner surfaces of a gall

**Figure 2:**
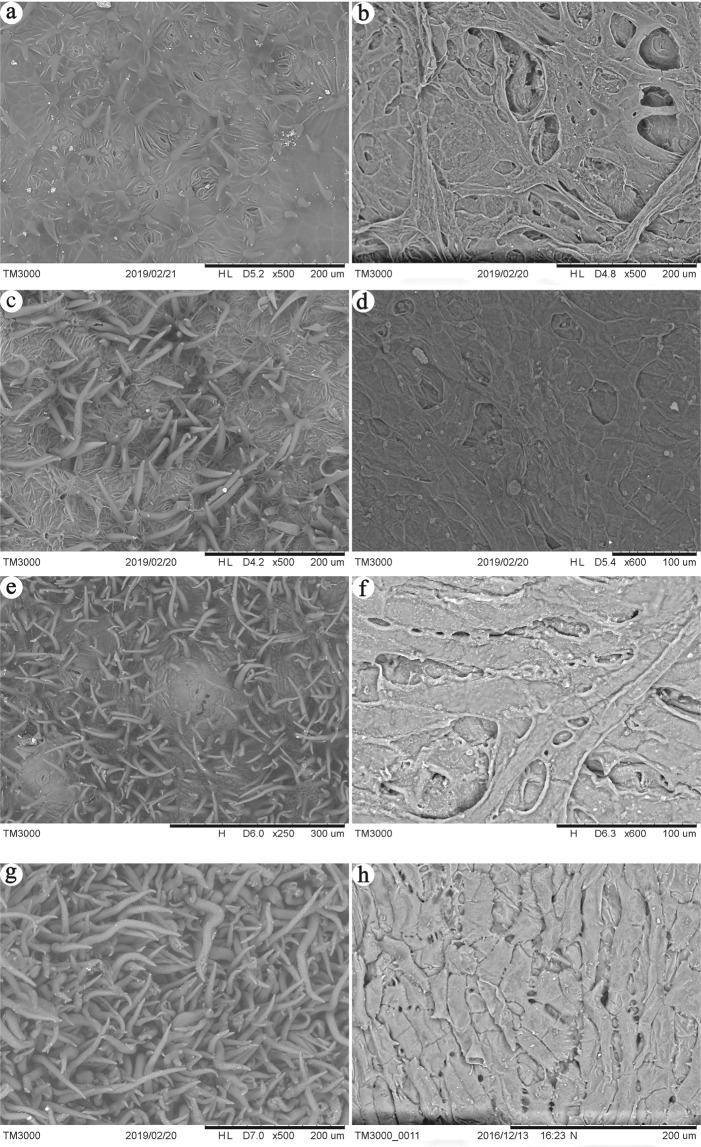
Scanning electron microscope images of the inner and outer surfaces of galls. (**a**) outer surface of the ensiform gall, (**b**) inner surface of the ensifom gall, (**c**) outer surface of the circular gall, (**d**) inner surface of the circular gall, (**e**) outer surface of the horned gall, (**f**) inner surface of the horned gall, (**g**) outer surface of the flower-like gall, (**h**) inner surface of the flower-like gall.

The outer surface of ensiform gall, circular gall and horned gall contains large number of tomentum that intersperse stomas, with a potential role in facilitating air exchange (Fig. 2a,c,e). The outer surface of flower-like gall covered with tomentum completely, so the stoma can’t be found (Fig. 2g). There are 85.53 ± 25.28/mm^2^ stomas and 233.68 ± 71.92/mm^2^ tomentum on the outer surface of the ensiform gall, 160.52 ± 40.03 /mm^2^ stomas and 514.83 ± 95.95/mm^2^ tomentum on the outer surface of the circular gall, 9.92 ± 1.88/mm^2^ stomas and 1314.92 ± 93.21/mm^2^ tomentum on the outer surface of the horned gall and 2817.61 ± 216.48/mm^2^ tomentum of flower-like gall (Table 2).

**Table 2:** Number of stoma and tomentum in galls.

	Ensiform gall	Circular gall	Horned gall	Flower-like gall
stoma (/mm^2^)	85.53 ± 25.28b	160.52 ± 40.03a	9.92 ± 1.88c	×
tomentum(/mm^2^)	233.68 ± 71.92d	514.83 ± 95.95c	1314.92 ± 93.21b	2817.61 ± 216.48a

30 galls were counted of each type. n = 30, *P < 0.05.

The inner surfaces of galls are irregular and uneven, which contain many holes (Fig. 2b,d,f,h).

**Figure 3:**
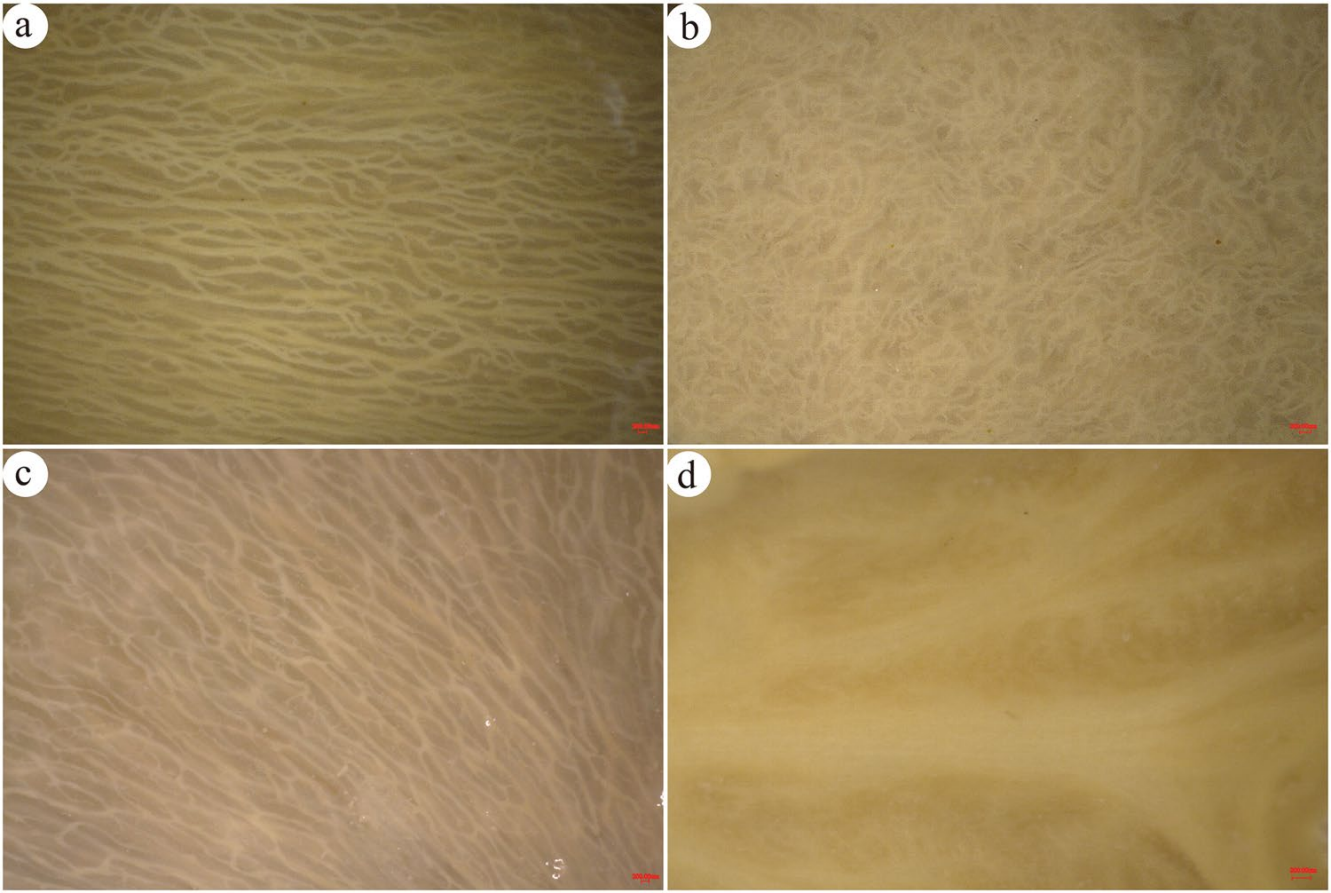
Images of the inner surfaces of galls. (**a**) the ensiform gall, (**b**) the circular gall, (**c**) the horned gall, (**d**) the flower-like gall.

The distribution of vascular bundles became more intuitive when gall walls were treated with a NAOH solution. Vascular bundles are criscrossed with branches in ensiform gall and horned gall, and generally distributed in parallel and extended from the base to the tip of a gall (Fig. 3a,c). Vascular bundles in circular gall distribute in the wall in a mess (Fig. 3b). The surface of flower-like gall can’t be observed the vascular bundles (Fig. 3d).

### The anatomical structure of ensiform gall

**Figure 4:**
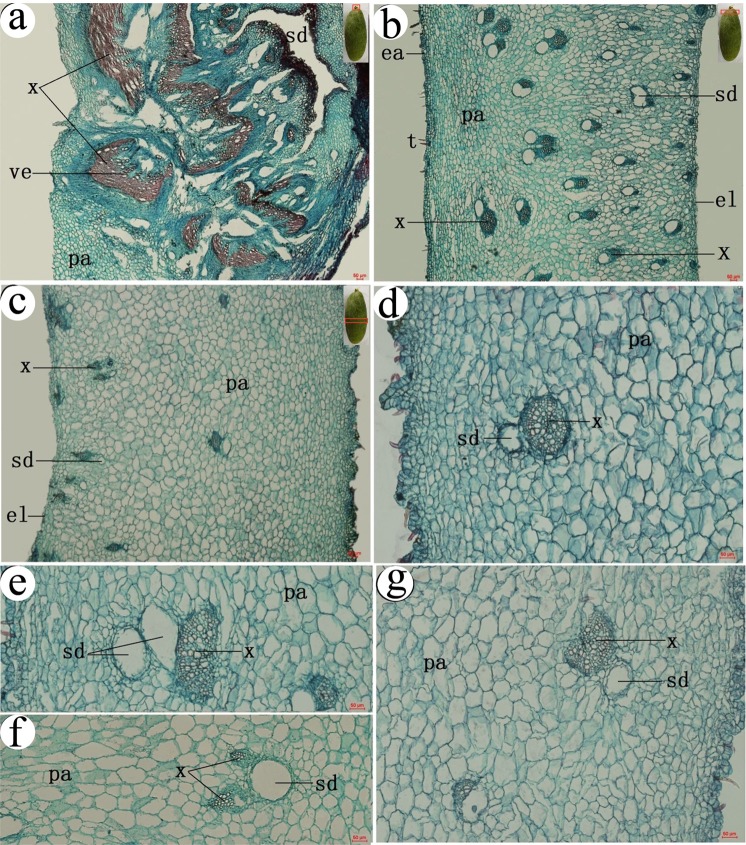
Anatomical structures of a ensiform gall. (**a**) inside base region (the stalk), (**b**) near the stalk, (**c**) gall body, (**d**) circular xylem, (**e**) half round xylem, (**f**) spindle xylem, (**g**) 3/4 circular xylem. t = tomentum, ea = epidermis-air, el = epidermis-lumen, vb = vascular bundle, pa = parenchyma, sd = schizogenous duct. x = xylem. The small pictures in the top right corner show the position of the sections which in the gall.

*S. chinensis* and *R. potaninii* is Anacardiaceae family, so the specialized schizogenous ducts of vascular bundles also found in galls^22^. There were similar multi-expanded xylems in stalk ensiform galls (Fig. 4a). A stalk not only serves as a mechanical support for the gall, but also provides a main channel for transporting nutrients. Parenchyma of outer layers became thicker with less vascular bundles in gall sections from the stalk towards the tip (Fig. 4c). There are many forms of xylems, including circular, half-round (Fig. 4d,e,g), and spindle ones (Fig. 4f). In most cases, one schizogenous ducts accompanies one xylem, but we did find two schizogenous ducts accompany one xylem in some cases (Fig. 4e).

### The anatomical structure of circular gall

**Figure 5:**
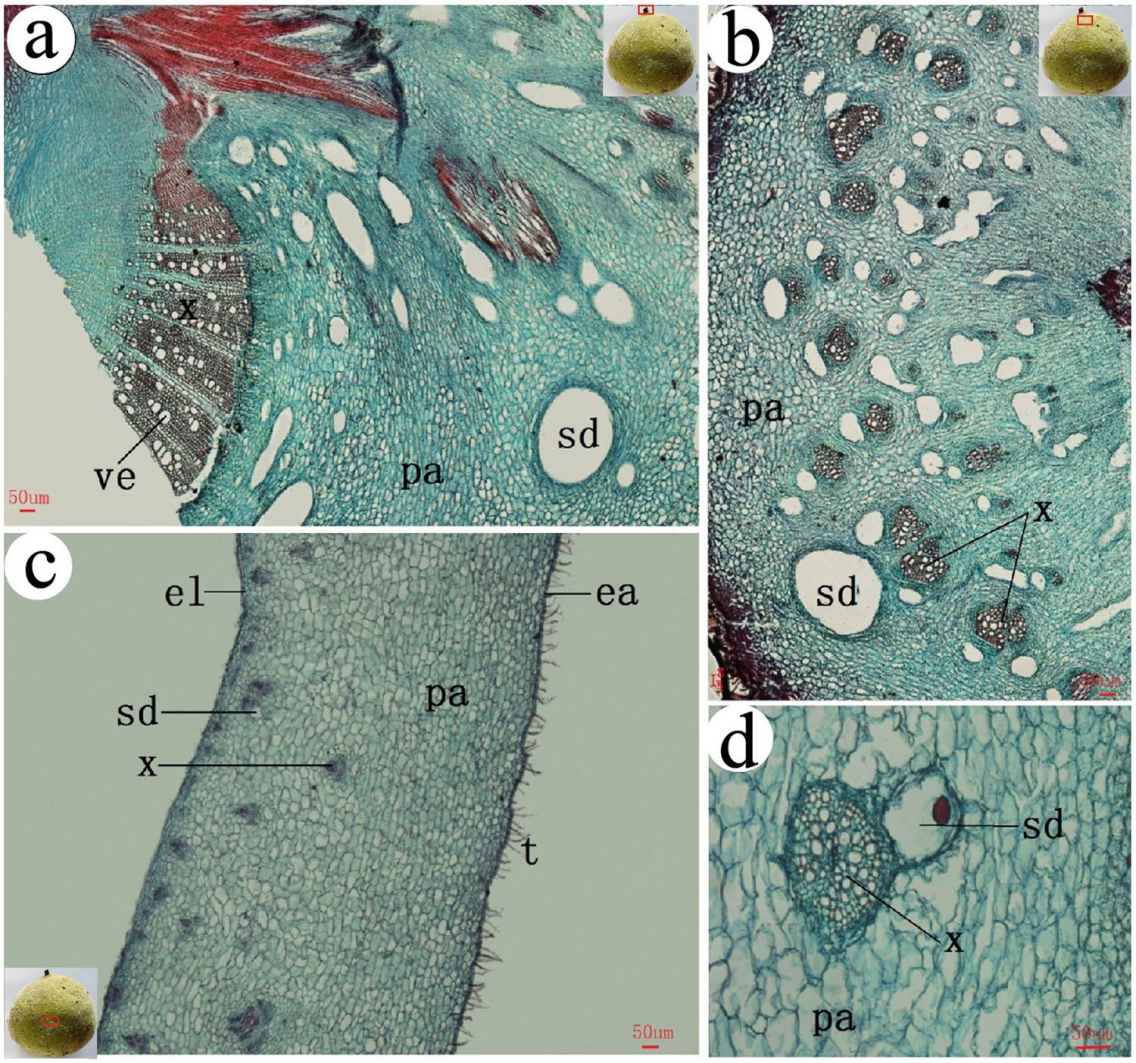
Anatomical structures of a circular gall. (**a**) the base (the stalk), (**b**) near the stalk, (**c**) gall body, (**d**) circular xylem. t = tomentum, ea = epidermis-air, el = epidermis-lumen, vb = vascular bundle, pa = parenchyma, sd = schizogenous duct, x = xylem. The small pictures in the top right corner show the position of the sections which in the gall.

There were abundant vascular bundles in the parenchymatous tissue. A stalk connected with the main vein. The xylem in the stalk structure was expanded greatly, and there were lots of tiny tubes distributed in the expanded xylem (Fig. 5a). There were many vascular bundles in the wall (Fig. 5b,c). We also found a small number of regular circular xylems in circular galls (Fig. 5d).

### The anatomical structure of horned gall

**Figure 6:**
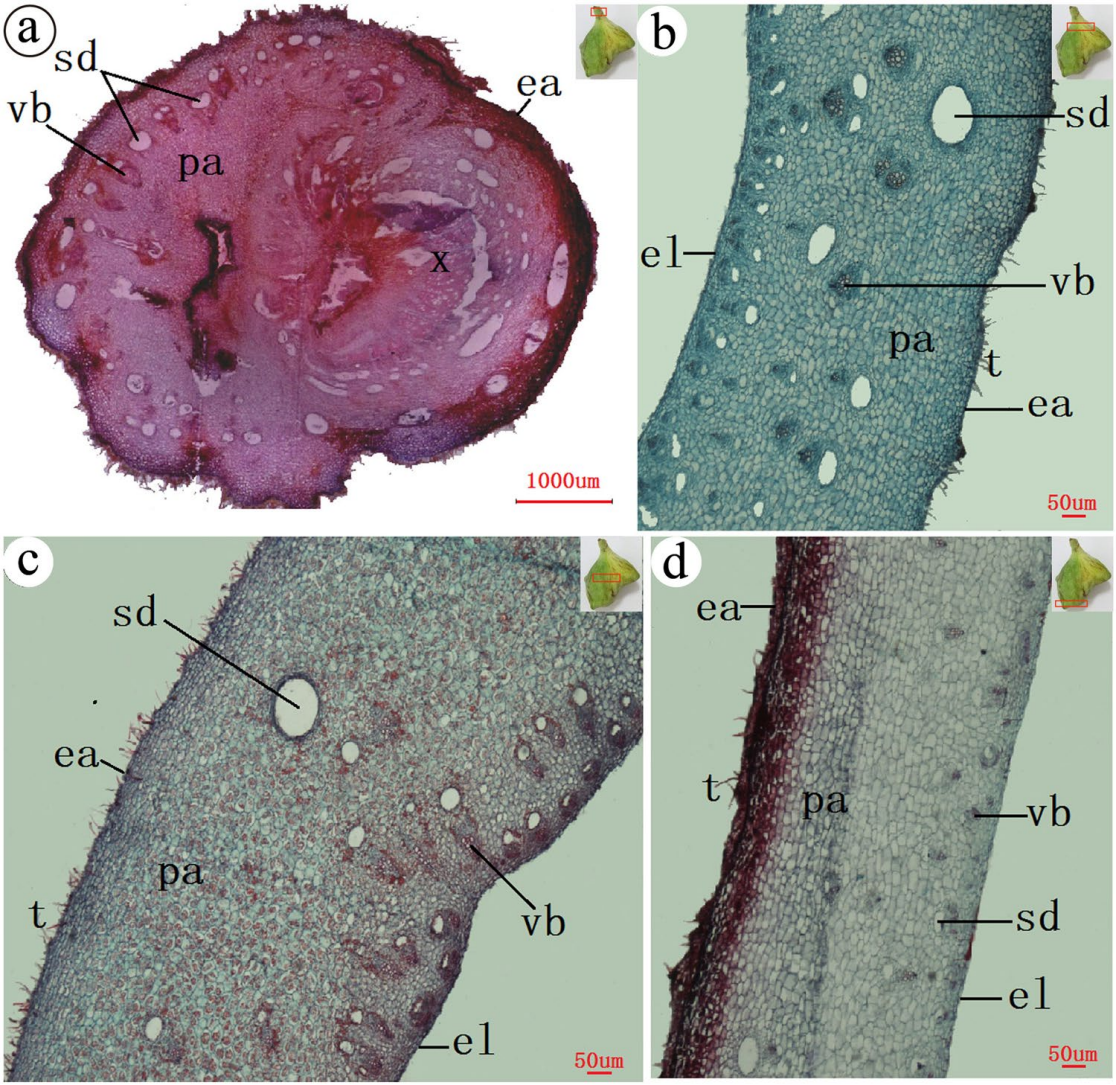
Anatomical structures of a horned gall. (**a**) the base (the stalk), (**b**) near the stalk, (**c**) the middle of the gall body, (**d**) far away from the stalk. t = tomentum, ea = epidermis-air, el = epidermis-lumen, vb = vascular bundle, pa = parenchyma, sd = schizogenous duct. The small pictures in the top right corner show the position of the sections which in the gall.

At the base of horned gall, a stalk connected with a rachis (Fig. 6a). There were abundant vascular bundles in the stalk (Fig. 6a). At the junction between the gall and rachis wing, xylem was found expanded greatly, occupied nearly one-third space of the gall base region. There were lots of tiny tubes distributed in the expanded xylem (Fig. 6a).

The distribution of vascular bundles was significantly different between the main body and the stalk base region of a gall (Fig. 6). There were more vascular bundles in the inner layers of a gall than in the outer layers (Fig. 6b–d). Parenchyma in outer layers became thicker with few vascular bundles in gall sections from the stalk base towards the tip. Vascular bundles were intertwined and mostly distributed in the inner layers.

Epithelial cells distributed in both outer and inner layers of a gall. In cross sections, there were granular contents distributed evenly throughout the gall wall (Fig. 6c).

### The anatomical structure of flower-like gall

**Figure 7:**
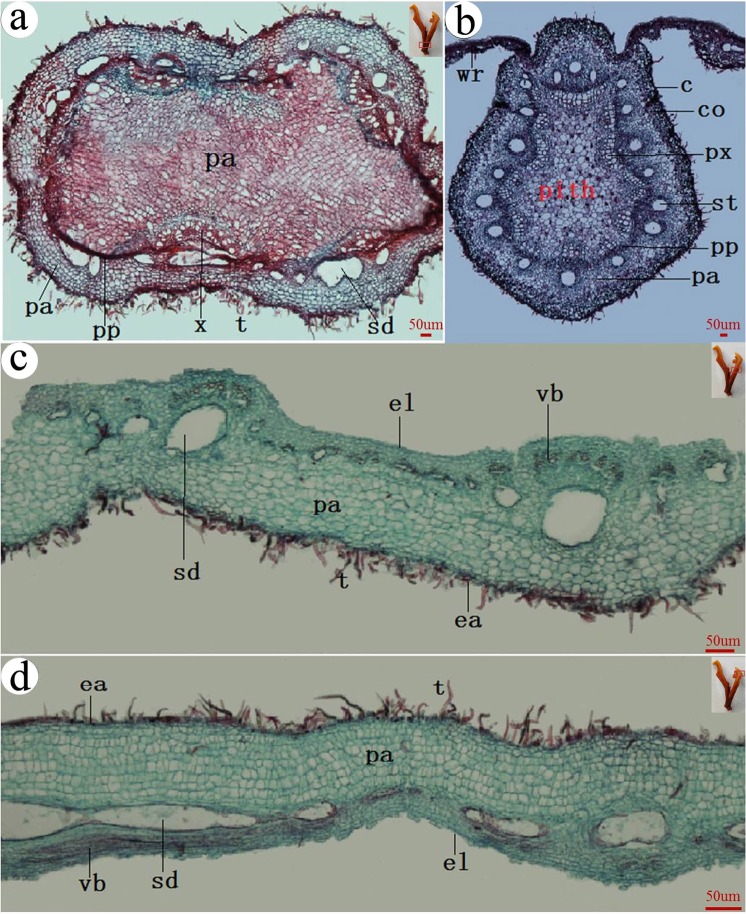
Anatomical structures of a flower-like gall. (**a**) the base (the stalk), (**b**) rachis, (**c**) near the stalk, (**d**) gall body. t = tomentum, ea = epidermis-air, el = epidermis-lumen, vb = vascular bundle, pa = parenchyma, sd = schizogenous duct. x = xylem. The small pictures in the top right corner show the position of the sections which in the gall.

Inside the stalk which was filled with parenchyma surrounded by a circular primary phloem (Fig. 7a). There were equally distributed xylem in the inner layer of the primary phloem, and there were irregular vascular bundles on the outer layer of the primary phloem (similar with sieve tube of rachis). Uniform parenchyma was found in the outer layer of the gall wall. The overall structure of a stalk of a gall was similar to that of a typical rachis (Fig. 7b).

Vascular bundles exhibited unique organization in flower-like galls. Major and minor schizogenous ducts were located near the stalk structure. Major schizogenous ducts were associated with multiple xylems (Fig. 7c). Schizogenous ducts become longer and thinner in regions which farther away from the stalk. Despite their locations, vascular bundles were distributed only in the inner region of the wall, and were arranged in stripes with no obvious intervals in the inner regions of the wall (Fig. 7d). Cellular constituents in the wall exhibited unique organization as well. The wall appeared split at schizogenous ducts - boundaries into separate parenchyma. Cells near the chamber were smaller than those in the outer regions of the wall (Fig. 7c,d).

### The size and quantity of schizogenous ducts among galls

We counted the diameter and the density of schizogenous ducts in different position of different galls. In the stalk, the biggest schizogenous ducts was found in outer layer of ensiform gall (13.21 ± 2.77 um), followed by horned gall (8.64 ± 3.85 um), flower-like gall (7.65 ± 4.55 um) and the circular gall (7.51 ± 2.85 um). The biggest of inner layer in the stalk was ensiform gall (11.91 ± 3.69 um), followed by horned gall (7.65 ± 2.46 um) and circular gall (7.30 ± 3.87 um). Schizogenous ducts close to the stalk in ensiform and horned gall are bigger than circular gall no matter in inner layer or outer layer. Schizogenous ducts in main body of ensiform and horned gall are bigger than circular gall too. The schizogenous ducts only distribute in outer layer in stalk and inner layer in other two positions, and bigger than other galls because which like banding (Table 3). The schizogenous ducts in stalk are much bigger than which nearby the stalk, and following which in the main body of the gall in all type of galls besides the flower-like gall (Table 3).

**Table 3:** Diameter of schizogenous ducts in galls.

		Ensiform gall	Circular gall	Horned gall	Flower-like gall
stalk (um)	outer layer	13.21 ± 2.77 aA	7.51 ± 2.85bA	8.64 ± 3.85bA	7.65 ± 4.55bA
	inner layer	11.91 ± 3.69 aA	7.30 ± 3.87bA	7.65 ± 2.46bA	×
near the stalk (um)	outer layer	6.66 ± 3.10aB	4.69 ± 1.23bB	6.92 ± 3.59 aA	×
	inner layer	4.01 ± 3.28bB	3.98 ± 2.03bB	4.67 ± 1.63bC	6.32 ± 1.22 aA
the main body (um)	outer layer	5.56 ± 2.54aB	3.33 ± 3.88bB	6.05 ± 2.34aB	×
	inner layer	3.21 ± 1.99bC	2.66 ± 1.51cC	3.31 ± 1.97bD	9.71 ± 9.80 aA

30 sections were measured in each position. n = 30, *P < 0.05. Capital letters represent the analysis result of different positions in one type gall (portrait orientation). Lowercase mean the result was analyzed among different galls (landscape orientation).

In the stalk, schizogenous ducts are the most in outer layer of horned gall (13.37 ± 1.71/mm^2^), followed by flower-like gall (12.88 ± 2.01/mm^2^), ensiform gall (11.32 ± 3.77/mm^2^) and the circular gall (10.03 ± 0.97/mm^2^). Sschizogenous ducts of the horned gall (12.47 ± 1.96/mm^2^) were more than ensiform (10.54 ± 2.30/mm^2^) and circular (9.36 ± 0.74/mm^2^), gall in the inner of the stalk. The density of schizogenous ducts in the inner layer is much more than schizogenous ducts which in the outer layer in all galls. Generally, the schizogenous ducts of horned gall are more than other galls (Table 4).

**Table 4:** The density of schizogenous ducts in galls.

		Ensiform gall	Circular gall	Horned gall	Flower-like gall
stalk (/mm^2^)	outer layer	11.32 ± 3.77 aA	10.03 ± 0.97bA	13.37 ± 1.71aB	12.88 ± 2.01 aA
	inner layer	10.54 ± 2.30 aA	9.36 ± 0.74bA	12.47 ± 1.96aB	×
near the stalk (/mm^2^)	outer layer	3.55 ± 0.756aD	2.88 ± 0.25aC	3.73 ± 2.49aD	×
	inner layer	7.01 ± 2.30bB	3.62 ± 1.11cC	16.77 ± 2.99 aA	7.67 ± 0.47bB
the body (/mm^2^)	outer layer	0.59 ± 0.06bE	1.24 ± 0.46aD	0.15 ± 0.27cE	×
	inner layer	6.14 ± 0.43bC	7.18 ± 1.38bB	9.28 ± 5.35aC	1.81 ± 0.84cC

30 sections were counted in each position. n = 30, *P < 0.05. Capital letters represent the analysis result of different positions in one type gall (portrait orientation). Lowercase mean the result was analyzed among different galls (landscape orientation).

## Discussion

**Figure 8:**
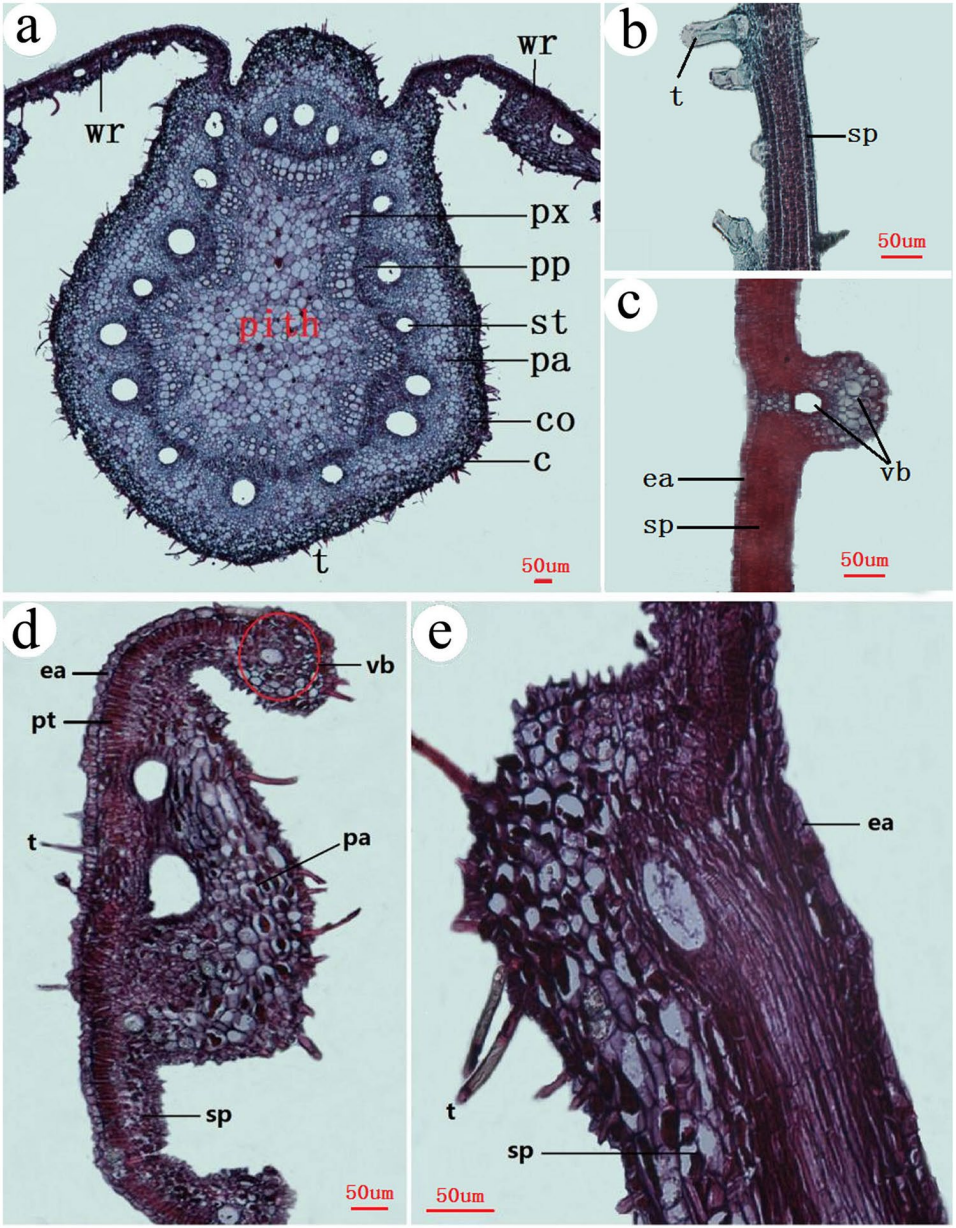
Cross sections of a rachis, leaf blade, rachis wing and hyperplasia rachis wing. (**a**) axillary bud. (**b**) leaf blade of *S. chinensis*. (**c**) leaf blade of *R. potaninii* Maxim. (**d**) rachis wing fed by fundatrix. (**e**) hyperplasia rachis wing. wr = winged rachis, px = primary xylem, pp = primary phloem, st = sieve tube, pa = parenchyma, co = collenchyma, c = cuticle, t = tomentum, ea = epidermis - air, vb = vascular bundle, pt = palisade tissue, sp = spongy parenchyma.

In this study, we compared structures of galls induced by four different aphid species. The structures of gall-inducing sites were distinctly different among these species. *N. shiraii* feeds and induces galls on axillary buds, which are of typical dicotyledon characteristics, including piths, primary xylem, primary phloem and collenchyma (Fig. 8a). *S. peitan* feeds and induces galls on or near the main vein of a leaf, whereas *K. r. rhusicola* feeds and induces galls on the petiole of a leaf. The leaf structures of both *R. chinensis* and *potaninii* are similar, with layered spongy parenchyma (Fig. 8b,c). *S. chinensis* feeds and induces galls on rachis wings, which consist of palisade, spongy parenchyma, vascular bundles, epidermal cells, and singular epicuticles (Fig. 8b,d).

Based on previous research, the gall parenchyma was distinct, taxonomy is not consistent with the histological characteristics of the galls, the identification of species based on microscopic features of galls is necessary^23,24^. In our study, the different feeding sites for specific aphid species could be the results of long co-evolution between the host plants and aphids. Leaf and rachis wing has thinner cuticle than axillary bud, and axillary bud has collenchyma. So aphids easier fed in leaf and rachis wing, which lead to *S. chinensis, S. peitan* and *K.r.rhusicola* had more survivor than *N.shiraii*. Flower-like galls induced by *N. shiraii* on axillary buds fall off the gall-inducing site easily (Fig. 1h). That’s probably why there are many branches of a gall to reduce gall weights. Horned galls induced by *S. chinensis* on rachis wings are steady mechanically because the wings are close to the main vein of a leaf (Fig. 1f). Circular galls induced by *S. peitan* are located individually on the main vein of a leaf (Fig. 1d) and ensiform galls induced by *K. r. rhusicola* are located individually on a petiole of a leaf (Fig. 1b). Therefore, both circular horned and ensiform galls are mechanically firm on gall sites.

Another important impact of gall locations is nutrient transport. Flower-like galls are far away from a leaf, which could be a disadvantage for nutrient transportation. Horned galls are next to the main vein of a leaf, which is apparently convenient for obtaining nutrients from both roots and leaves. However, multiple galls located on a single rachis wing (Fig. 1f) could result in nutritional competition. Circular galls and ensiform galls are on or next to the main vein of a leaf and one leaf holds only one gall. This arrangement is beneficial in terms of nutrients to aphids.

**Figure 9:**
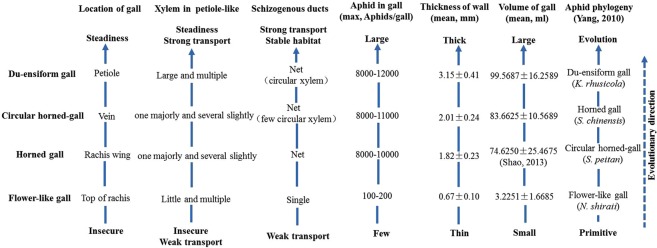
Evolution analysis.

In the stalk structure of each gall type, there were abundant vessels and normal or expanded xylems. The number and size of xylem structure differed in different gall types. In ensiform galls, there were multiple expanded xylems in the base regions (the stalk structure). In circular galls and horned galls, there was only one majorly expanded xylem and several slightly expanded xylems. In flower-like galls, there were equally distributed, normally sized xylems in the inner region of the primary phloem. The numbers and sizes of xylems correlated with the ability of a gall to support the sizes of aphid populations (Fig. 9). This can be seen that all xylems were expanded in ensiform galls, which are bigger in sizes and support more aphids. In comparison, there was no expanded xylem in the flower-like galls, which host less aphids. Similar situations were also found with the presence of vascular bundles. Our overall data support the postulation that the evolution direction of galls favors steadier affirmation of galls onto host plants and higher efficiency for nutrient transport. In the gall types we have examined, the evolution trail has been from flower-like galls to horned galls, to circular galls, and to ensiform galls (Fig. 9).

The rough surface of the galls were likely evolved to provide a favorable environment for aphids, with rough lumen easy for aphids to adhere, pores facilitate exchanges of air, nutrients and metabolites. The distribution of vascular bundles in a gall is interesting. In flower-like galls, vascular bundles were arranged in a monolayer in the innermost region of the wall. In horned, circular horned-, and ensiform galls, a network of vascular bundles was found with multiple branches distributed irregularly with twists and turns, providing a stable circulation system. This arrangement provides expanded areas for aphids to feed and for exchanges of air, nutrients, and metabolites (Fig. 9). It has been hypothesized that gall structures are diversified in response to selection pressure for the enhancement of the surface area available for gall inducers to feed^25^. The array of vascular bundles in parallel from the base to the tip of a gall also provides advantage to ensure nutrients to be directly and rapidly transported from the base of a gall to the rest of the gall tissues. The network of vascular bundles has also been differentiated to preserve water inside galls and protect galls from microbial and predator invasions. The diameters of schizogenous ducts and other pore structures become smaller from inner gall tissues towards outer layers. The reduced sizes of pores may prevent water evaporation from galls. Our observations are consistent with previous reports that horned galls have the ability to adjust temperature and maintain moisture under different environmental conditions and during day/night transitions. In fact, internal humidity of a gall is maintained at 99.9–100% during the whole gall growth period and dankness was in favour of aphid^26^. Importantly, we found lots of circular and semicircular xylems in ensiform galls, which would provide more nutrition in limited spaces. Though rare, circular xylems were found in circular galls.

The size of galls correlated with the thickness of gall walls. We propose that the evolutional trail of galls has been from small to big (Fig. 9). A bigger space in a gall can accommodate more aphids, and a thicker wall is also beneficial to prevent aphids from natural enemies and store more chemicals such as tannins, which may protect gall tissues from herbivores^8,27^. More vascular bundles distributed in thicker walls facilitate more efficient transport of photoassimilates.

The evolutionary trail of the molecular phylogeny among the four aphid species was ensiform gall (*K.r.rhusicola*), horned gall (*S. chinensis*), circular gall (*S. peitan*), and flower-like gall (*N.shiraii*)^28^ (Fig. 9). Even though the evolutionary trail is almost same as the results from the galls’ location and anatomical structures, we think the circular gall (*S. peitan*) is more evolution than horned gall (*S. chinensis*). Circular gall are infrequent in fields and unstable on a leaf of a plant although *S. peitan* gets nutrition easily. We speculate that circular gall may be only occasional and transitional in the process of evolution. Our results indicated that gall shapes and structures of the wall are not only related with gall-inducers but also with the type of host plants, particularly the location of a gall on a host plant. Gall shapes, anatomical structures and locations on host plants may reveal evolutional trail in a gall-aphid relationship.

## Materials and Methods

### Sample collection and treatment

The samples which we used were come from the 5–6 year-old trees. And the height of the tree is about 2–3 meters. The random samples of galls (120 days after the galls formed) were collected from the different growth stage trees which come from the wild natural forests in Yan Jin county, Yunnan province, China (104.377704 E, 28.117765 N). Two or three galls were collected in each tree and collection contains more than 30 wild trees. The thickness was measured of fresh galls, then different positions (the stalk, close to the stalk, the junction of horizontal axis and the vertical axis of the galls, far away from the stalk) of 45 galls of each type were cut into 2–3 mm pieces and fixed immediately in FAA (formaldehyde 5 ml, acetic acid 5 ml, and 70% ethyl alcohol 90 ml) apartly for 5 days, only one piece was collected in the same position of single gall. Then stored in 70% ethyl alcohol for paraffin sections.

### Thickness of the fresh galls

**Figure 10:**
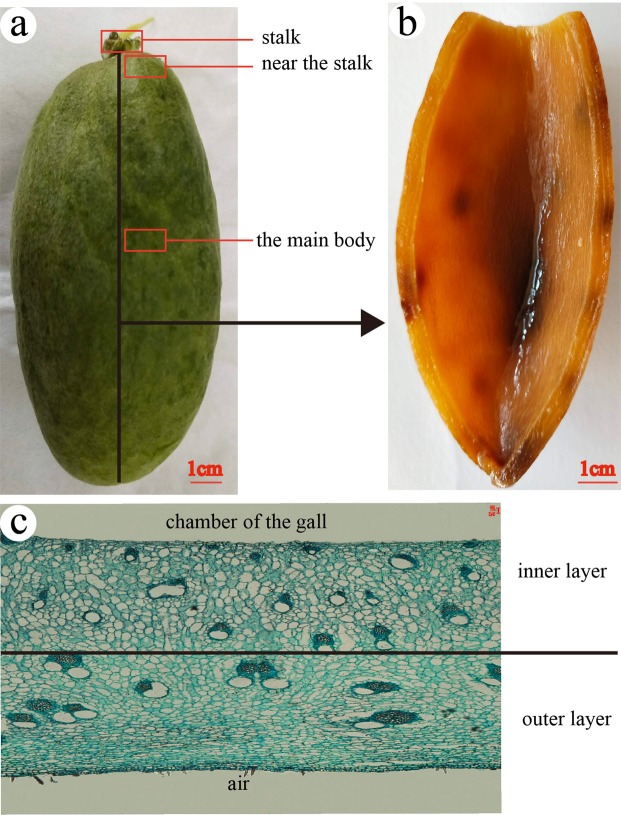
The detailes of methods. (**a**) the cutting position for measuring the thickness and three positions where measured the diameter and quantity of schizogenous ducts. (**b**) the view of an opening ensiform gall, (**c**) the inner layer and the outer layer of paraffin section.

The fresh galls were cut open from the vertical axis of the galls, then detected four positions randomly (Fig. 10a,b). 45 galls were measured each kind of gall.

### Paraffin sections

Selected the sample randomly, the samples which come from the same position of one type of the gall was detected more than 30 pieces. Samples were dehydrated in an ethanol series (70% ethyl alcohol for 1 h, 80% ethyl alcohol for 30 min, 90% ethyl alcohol for 20 min, 95% ethyl alcohol for 10 min, 100% ethyl alcohol for 5 min). Then bathed the samples in ethyl alcohol: dimethylbenzene = 1:1 sulotion for 1 h, alcohol: dimethylbenzene = 1: 3 sulotion for 30 min, dimethylbenzene for 1 h, dimethylbenzene: paraffin = 1:1 for 1 h, dimethylbenzene: paraffin = 1: 3 for 1 h, paraffin for 1 h. The processed samples were embedded in paraffin, and 25-um-thick sections were made using a rotary microtome (Leica RM2126RT, Germany), the sections were incubated for overnight at 50 °C and were deparaffinized and stained with safranin and fast green^29^. More than 30 galls which come from the different trees were measured by paraffin section.

The paraffin sections of galls were divided into two equal sides from the cross section, diameter and the number of schizogenous ducts in outer layer and inner layer (Fig. 10c) of three positions (Fig. 10a) were counted under an optic microscope (Nikon Eclipse Boi, DS-Fi1, Japan). 30 sections were measured of each position.

### The inner and outer surface of the galls

For Scanning Electron Microscope (SEM) observation, mature galls were fixed immediately in 2.5% glutaraldehyde for one hour and coated with gold. The surface of epidermal lumen and epidermal hairs of galls were examined under a scanning electron microscope (Tabletop Microscope 3000, Japan). The density of stoma and tomentum in three random positions were counted of single gall. 30 galls were counted of each type.

For 3-dimensional microscopic observations, mature galls were fixed immediately in 50% NAOH for 48 hours and then the surface of epidermal lumen was examined under a 3-dimension microscope (MSD-VHX1000, Japan).

### Data analysis

Nonparametric test was used for analysis by spss 17.0. We detected the mean value and single factor analysis was used. The data of every position were variable. P-value < 0.05.
